# Multimodal collaborative UAV framework for single rubber tree parsing

**DOI:** 10.1016/j.plaphe.2026.100227

**Published:** 2026-05-23

**Authors:** Weiqi Yin, Jie Zhang, Hengrui Wang, Yuanyuan Zhang, Jialei Zhan, Jialang Liu, Hui Lin, Jiangquan Zeng, Wentao Peng, Guoxiong Zhou, Huaiqing Zhang, Xiangjun Wang

**Affiliations:** a Central South University of Forestry and Technology, Changsha, Hunan, 410004, China; b Rubber Research Institute, Chinese Academy of Tropical Agricultural Sciences, Haikou, 571101, China; c Laboratory for Big Data and Decision, National University of Defense Technology, Changsha, 410073, China; d Chinese Academy of Forestry, Beijing, 100091, China

**Keywords:** Rubber tree, LiDAR point cloud, Individual-tree segmentation, Multimodal fusion

## Abstract

Accurate individual-tree segmentation in rubber tree plantations is essential for resource inventory and agro-forestry management, but it remains difficult in densely planted stands because adjacent crowns often overlap, point density varies markedly along the vertical canopy structure, and tree morphology differs across growth stages. To address these challenges, we introduce MDA-SegNet, a multimodal point-cloud instance segmentation framework designed for densely planted rubber plantations. The Multimodal Deformable Encoding (MDE) module integrates top-view orthophoto cues with light detection and ranging (LiDAR) geometry, providing complementary crown-boundary information for more consistent separation of adjacent trees. The Z-Order Selective Mamba (ZOS-Mamba) module converts three-dimensional spatial structures into locality-preserving sequences and models long-range vertical dependencies under uneven point-density distributions. The Adaptive Lemming Optimization Clustering (ALOC) module further refines instance separation by adapting to canopy overlap and morphological heterogeneity. Experiments were conducted on our self-constructed rubber tree dataset (RT-Set) and five public forest datasets. Compared with current state-of-the-art models, MDA-SegNet achieved higher F-score and mean Intersection over Union (mIoU), showing stronger robustness and cross-domain generalizability. On RT-Set, it obtained an F-score of 87.32% and a Recall of 82.90%, with improvements of 6.86 and 7.24 percentage points, respectively. These results indicate that MDA-SegNet can provide reliable individual-tree segmentation for dense rubber plantations.

## 1 Introduction

Natural rubber is an important economic crop in tropical regions, but the decline in natural rubber prices has reduced farmers’ income and gradually weakened the available labor force for rubber plantation management [1]. At the same time, recent advances in UAV platforms and lightweight sensing systems have made it feasible to acquire high-resolution LiDAR and multi-source remote sensing data with reduced field labor [2,3]. UAV-derived point clouds have therefore become an important data source for individual-tree segmentation, structural parameter extraction, and plantation-scale growth monitoring [4,5]. For rubber plantations, accurate individual-tree segmentation can provide tree location, crown structure, and growth-related information, which is essential for resource inventory, phenotypic observation, and precision plantation management.

Early studies on individual-tree analysis mainly relied on geometric priors and handcrafted rules. For example, individual tree attributes have been estimated from airborne laser point clouds using machine learning-based feature representations [6], while Layer Stacking and Treeiso attempted to isolate individual trees by exploiting vertical stratification or 3D graph structures [7,8]. These methods do not require large-scale labeled datasets and are effective in relatively regular forest structures. However, their performance often decreases when adjacent crowns overlap heavily, when point density changes sharply from the upper canopy to the lower trunk, or when crown boundaries are not clearly separated. In such cases, over-segmentation, under-segmentation, and unstable crown delineation may further affect structural parameter estimation. Previous studies on individual-tree attribute estimation also indicate that stable crown delineation is a prerequisite for reliable stem and crown parameter prediction [9]. These limitations suggest that purely geometry-prior-based unsupervised methods are insufficient for dense rubber plantation scenes, where stronger feature representation and instance discrimination are required.

With the development of deep learning, forest point cloud segmentation has gradually shifted from handcrafted geometric rules toward data-driven representation learning. Recent studies have explored cross-platform robustness, supervised 3D crown segmentation, and end-to-end forest point cloud segmentation. Henrich and van Delden investigated the possibility of training general deep learning models for tree instance segmentation across heterogeneous laser-scanning datasets [10]. TreeisoNet further provided a unified supervised framework for 3D crown segmentation across airborne, UAV-borne, and terrestrial laser scans [11], while ForestFormer3D explored an end-to-end segmentation framework for forest LiDAR point clouds [12]. These studies show the potential of deep learning for forest point cloud segmentation, but their target scenes are generally broader forest environments rather than dense rubber plantations with overlapping crowns, sparse lower trunks, and multi-stage structural heterogeneity.

For rubber plantations, deep learning-based segmentation and phenotyping have also received increasing attention. Wang et al. demonstrated the feasibility of individual rubber tree segmentation under terrestrial LiDAR conditions using a Faster R-CNN-based pipeline [13]. RTreeNet improved fine-scale individual rubber tree segmentation from UAV LiDAR point clouds by enhancing boundary awareness and suppressing ground-noise interference [14]. RsegNet further combined feature learning and dual-channel clustering to improve individual rubber tree segmentation and structural parameter extraction from UAV LiDAR point clouds [15]. More recently, Gao et al. proposed DDB-YOLO, a lightweight deep learning model for individual rubber tree crown segmentation from UAV imagery, aiming to address dense crown overlap, irregular crown shapes, and background interference in vegetation index derivation [16]. Tan et al. developed a UAV and handheld LiDAR co-registration framework for fine phenotyping of rubber plantations with complex canopies, showing the importance of multi-source LiDAR sensing for accurate structural characterization [17]. These studies indicate that rubber plantation monitoring is moving toward more precise individual-tree-level analysis. However, most existing rubber-tree studies still focus on either image-based crown delineation, multi-source data registration, or single-modal point-cloud segmentation. The explicit integration of 2D crown-boundary guidance, density-aware 3D spatial continuity modeling, and adaptive instance-level clustering for UAV LiDAR-based individual rubber tree segmentation remains insufficiently explored.

Meanwhile, multimodal feature fusion and lightweight deep networks have been widely explored in remote sensing and plant phenotyping tasks. Existing studies have shown that gated feature fusion, meta-encoding, transformer-based segmentation, and multimodal open object detection can improve feature representation under complex agricultural or remote sensing scenes [[18], [19], [20], [21]]. Lightweight point cloud networks have also been developed for plant organ segmentation, indicating the importance of efficient structural representation in agricultural point clouds [22]. In forest health monitoring, recent studies on pine wilt disease detection have further shown the value of lightweight feature fusion networks and Mamba-attention-based UAV imagery models for fine-grained forestry applications [[23], [24], [25]]. However, these studies mainly focus on image-level classification, disease detection, semantic segmentation, or general feature fusion. They do not directly address the instance-level separation of densely planted rubber trees from UAV LiDAR point clouds, where 2D boundary cues, 3D spatial continuity, and adaptive clustering need to be considered simultaneously.

Therefore, although recent forest point cloud and multimodal learning studies have achieved considerable progress, several limitations remain when they are applied to dense rubber plantations. First, existing multimodal fusion strategies usually combine heterogeneous features at a general semantic level, but they rarely use crown-boundary-oriented 2D cues as explicit guidance for 3D point-wise instance separation. Second, many point cloud segmentation networks rely on local neighborhood aggregation, sparse convolution, or global attention, but they do not explicitly model the spatial continuity from dense upper canopies to sparse lower trunks under strong vertical density gradients. Third, fixed-scale feature extraction or fixed-parameter clustering is often insufficient for rubber plantations with coexisting young and mature trees, where crown size, branch density, and stem morphology vary substantially. These limitations motivate a framework that can jointly exploit 2D boundary guidance, 3D structural continuity, and adaptive instance-level separation.

Accordingly, three challenges remain critical for individual rubber tree segmentation.

1. Ambiguous crown contours. In densely planted rubber plantations, adjacent crowns often overlap and form continuous canopy surfaces. Since LiDAR point clouds mainly describe geometric structure and lack the rich texture cues available in optical imagery, points around crown-transition zones may have similar spatial distributions, making individual boundaries difficult to distinguish [26].
2. Uneven vertical density gradients. Rubber trees usually contain dense foliage in the upper canopy but sparse returns around trunks and lower branches. Similar canopy–trunk density contrasts have also been reported in tree-related LiDAR and mobile laser scanning studies [27]. This vertical imbalance can cause models to overfit canopy-dominant features while under-representing lower structural components.
3. Structural heterogeneity across growth stages. Rubber trees at different developmental stages differ in crown size, branch architecture, and stem morphology. Such ontogenetic structural variation has been widely observed in tree architecture studies [28]. A fixed-scale representation may therefore fail to accommodate both small young trees and large mature trees in the same plantation.

Several studies have attempted to address crown-boundary ambiguity in forest or tree point clouds. Semantic–instance joint segmentation, end-to-end LiDAR-based tree detection, and adaptive sampling strategies have been used to improve object discrimination under complex backgrounds [[29], [30], [31]]. However, most of these methods are designed for general mobile mapping scenes, urban trees, or generic 3D scene understanding. They do not explicitly introduce 2D crown-boundary cues into 3D point-wise feature learning. As a result, their ability to separate adjacent rubber tree crowns remains limited when crown-transition zones are heavily interlaced.

Uneven point-density distributions have also been investigated from different perspectives. ALS–TLS registration, density-gradient-based point cloud denoising, and geometric regularization have been explored to improve structural consistency under heterogeneous sampling conditions [[32], [33], [34]]. These methods demonstrate that density variation can strongly affect structural representation. Nevertheless, they mainly focus on registration, denoising, or urban reconstruction, rather than instance-level tree separation. Therefore, they provide limited support for modeling the dense-canopy and sparse-trunk structure commonly observed in UAV LiDAR rubber tree point clouds.

For multi-scale structural variation, hierarchical transformers, dual-attention transformer networks, and multi-scale attention models have been proposed to improve feature representation across different object sizes [[35], [36], [37]]. These methods enhance contextual modeling to some extent, but many of them still depend on predefined scale hierarchies or general feature aggregation schemes. In multi-aged rubber plantations, crown size, branch density, and stem morphology may vary substantially among neighboring trees. A more adaptive instance-level separation strategy is therefore required to accommodate such age-related structural heterogeneity.

To address these challenges, we propose MDA-SegNet, a multimodal deep learning framework for individual rubber tree segmentation and structural parameter extraction from UAV LiDAR point clouds. The main contributions of this study are summarized as follows.

1. We propose a multimodal boundary-guided feature enhancement module, named MDE, to address the weak boundary discrimination of geometry-only LiDAR representations in dense rubber plantations. Unlike general 2D–3D feature fusion strategies, MDE introduces structured 2D crown-boundary cues into 3D point-wise feature learning, thereby providing additional guidance for separating adjacent and partially occluded crowns.
2. We design a Z-order and Selective Mamba module, named ZOS-Mamba, to model the spatial continuity of rubber tree point clouds under uneven vertical density distributions. By combining Z-order spatial serialization with selective state-space modeling, ZOS-Mamba captures long-range structural dependencies from dense upper canopies to sparse lower trunks, reducing excessive reliance on local neighborhood aggregation.
3. We introduce an adaptive local object clustering module, named ALOC, for instance-level crown separation under multi-scale structural heterogeneity. Different from fixed-parameter clustering strategies, ALOC adjusts the clustering process according to local structural features, improving the separation of rubber trees with different crown sizes, branching patterns, and growth stages.
4. We evaluate MDA-SegNet on a self-constructed UAV LiDAR rubber tree dataset and two public forest point-cloud benchmarks, including five regional subsets from FOR-instance and the NIBIO_MLS dataset. Extensive comparisons with mainstream instance segmentation models and module-wise ablation studies demonstrate the effectiveness of the proposed multimodal boundary guidance, density-aware spatial modeling, and adaptive instance clustering under complex plantation conditions.

## 2 Materials

### 2.1 Study area

**Fig. 1:**
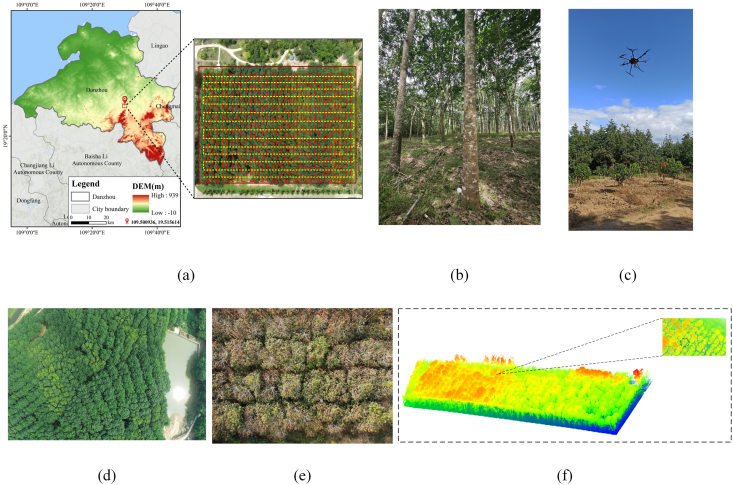
(a) Geographic location of the study site in Danzhou, Hainan Province, overlaid with the Digital Elevation Model (DEM) showing topographic variations; (b) Ground survey photograph revealing the vertical stratification and regular spatial layout of the rubber plantation; (c) The data acquisition platform: a DJI M300 RTK UAV mounted with the OriNav CBI-Lite LiDAR system; (d)–(e) Aerial orthophotos captured during the flight from different sampling zones; (f) The resulting raw 3D point cloud and its cross-sectional profile, effectively capturing the vertical gradient from the tree crown to the understory with high point density and sharp boundaries.

This study was conducted in a rubber plantation experimental site in the northwestern part of Danzhou, Hainan Province, China (19°31′50.59′′N, 109°28′52.62′′E), under the administration of the Chinese Academy of Tropical Agricultural Sciences (Fig. 1(a)). The region has a typical tropical monsoon climate, with an average annual temperature of 22.5–25.6 °C, annual precipitation of 900–2400 mm, and annual sunshine duration of 1780–2600 h. Under these climatic conditions, the vegetation is dominated by rubber trees (*Hevea brasiliensis*), and long-term plantation management has produced relatively regular planting patterns and canopy organization.

Although the plantation shows a generally regular stand structure due to management practices, local structural variation is still present. Frequent typhoon disturbances associated with the monsoon climate, seasonal phenological changes, and differences in local terrain can affect crown morphology, canopy closure, and vertical vegetation distribution. As shown by the Digital Elevation Model (DEM) in Fig. 1(a), the study site also exhibits topographic variation, which may further influence stand structure and point-cloud distribution. These conditions provide a representative and challenging scenario for evaluating three-dimensional individual crown segmentation in managed rubber plantations.

### 2.2 Data acquisition

The LiDAR data were collected on March 5, 2023, using a DJI M300 RTK UAV (Shenzhen, China) equipped with a CBI-Lite LiDAR system from OriNav (Chengdu, China) (Fig. 1(c)). The system supports 360° panoramic scanning and dual-echo acquisition, with a measurement range of 0–120 m, an accuracy of 5 mm, a repeatability of 10 mm at 30 m, and a maximum point acquisition rate of 640,000 points per second. It is also equipped with a 20 MP RGB camera with an 84° field of view, and the total system weight is approximately 1.28 kg.

To ensure spatial uniformity and high-quality reconstruction of the canopy structure, the UAV mission was conducted using a double-grid flight route over the representative sampling area (Fig. 1(a)). The flight altitude was 80 m above ground level, corresponding to approximately 50–60 m above the canopy. The average flight speed was 6 m/s, with a 10 m spacing between flight lines and 65% lateral overlap. The resulting point cloud reached a maximum density of 10,667 points/m^2^, providing detailed representation of canopy boundaries and internal structural features. The lower-left inset in Fig. 1(a) shows one representative sample plot within the study area, rather than the entire study region.

The sampling design was determined according to the structural characteristics of rubber plantations and the requirements of subsequent semantic and instance segmentation. Rubber trees in managed plantations are generally arranged in rows, with relatively regular planting intervals, similar crown shapes, and distinct vertical stratification from the upper canopy to the lower stem region. The dual-echo LiDAR acquisition helped capture both canopy surface returns and partial internal structural information, thereby improving the representation of vertical canopy organization. Based on these characteristics, representative stands with relatively low understory disturbance and typical rubber tree architecture were selected as the main acquisition areas. This sampling strategy improved the structural representativeness of the dataset and provided a reliable data basis for subsequent point-cloud modeling and individual-tree segmentation.

Fig. 1 summarizes the data acquisition workflow from field observation to point-cloud generation. The ground survey image in Fig. 1(b) shows the vertical stratification and regular spatial layout of the rubber plantation, confirming its suitability for structural modeling. The aerial images captured during the UAV flight (Fig. 1(d) and (e)) provide reference information for subsequent preprocessing steps, including region selection and boundary calibration. The raw 3D point cloud and its cross-sectional profile (Fig. 1(f)) further illustrate the vertical gradient from the crown layer to the understory. The acquired data exhibit high point density and clear structural boundaries, making them suitable for terrain normalization, semantic and instance annotation, and single-tree structural modeling.

### 2.3 Data pre-processing

**Fig. 2:**
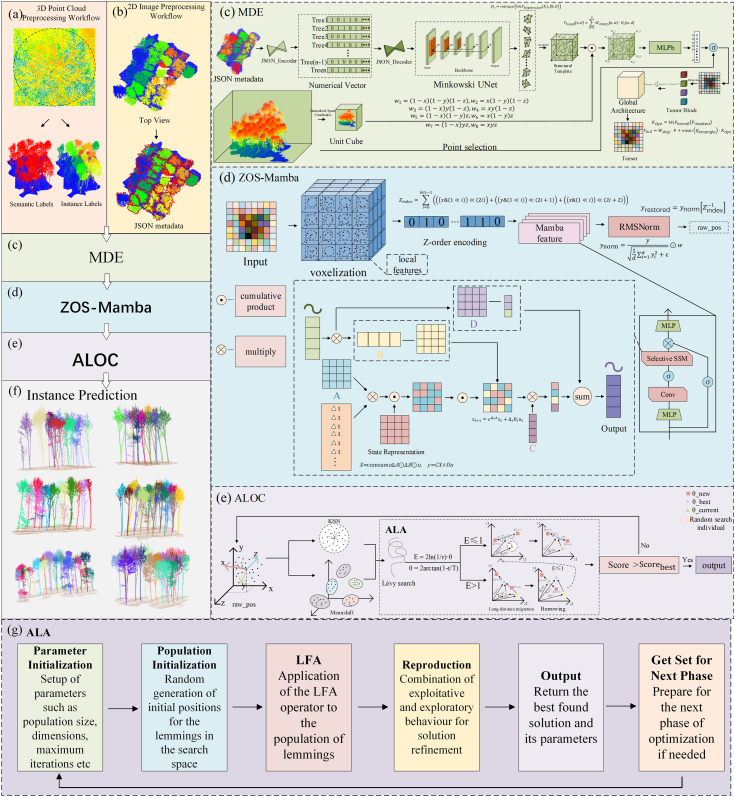
(a) 3D point cloud preprocessing workflow; (b) 2D image preprocessing workflow; (c) Multimodal Deformable Encoding (MDE); (d) Z-order and Selective Mamba (ZOS-Mamba); (e) Adaptive Lemming Optimization Clustering (ALOC); (f) Instance Prediction; (g) Adaptive Lemming Algorithm (ALA).

High-quality point clouds are essential for preserving complete tree structures and supporting multi-scale feature representation. Therefore, a structured preprocessing and label-generation pipeline was established for semantic and instance-level modeling. The pipeline included point-cloud cleaning, individual-tree extraction, semantic and instance annotation, and the generation of corresponding 2D annotations. As shown in Fig. 2, these steps provided a consistent data basis for subsequent cross-modal feature learning.

To generate 3D supervised data for multimodal learning, individual tree structures were extracted and annotated from the normalized canopy point clouds. The procedure began with top-view contour pre-screening (Fig. 2(a)), where candidate individual trees were identified according to crown boundaries and inter-tree spacing. The boundaries were then manually refined in CloudCompare from the top, side, and front views. During this process, points that did not belong to the target tree were removed through visual inspection, especially in canopy-overlapping regions. Local point-density differences and geometric continuity were also considered to preserve complete tree morphology and maintain clear individual boundaries.

Based on the refined individual-tree point clouds, spatially adjacent and morphologically consistent tree points were organized into annotation units. Each point was assigned two types of labels. The first was a semantic label, which distinguished rubber tree crowns of interest from the background. In this study, crown points were labeled as 1 and background points as 0. The second was an instance label, in which each rubber tree was assigned a unique identifier. Different tree instances were visualized using distinct colors to facilitate manual checking and quality control. This annotation strategy provided both category-level supervision and instance-level structural information for 3D point-cloud learning.

To support multimodal feature fusion, corresponding 2D annotations were also generated for the image branch. A top-view projection image was produced from the normalized point cloud, and the crown boundary of each rubber tree within the corresponding area was annotated using LabelMe (Fig. 2(b)). All crown annotations were stored as closed polygons and uniformly converted into JSON files. These 2D annotations preserved the relative spatial relationships of individual crowns in the top-view plane and provided boundary-related semantic cues for subsequent 2D–3D feature alignment.

It should be noted that Fig. 2 illustrates one representative spatial block among the 36 blocks used in this study. The remaining blocks were processed using the same preprocessing and annotation pipeline, but they differed in canopy density, tree age, phenological status, and local structural complexity. The complete dataset was divided into 23 training blocks, 8 validation blocks, and 5 testing blocks. This partition increased the structural variability of the dataset and enabled a more reliable evaluation of model performance under different plantation conditions.

To ensure annotation quality, all semantic and instance labels were checked through multiple rounds of manual verification. Only annotations that satisfied the predefined quality-control criterion, with more than 95% labeling consistency, were included in the final dataset. After quality screening, 600 rubber tree individuals with complete structures and diverse morphologies were retained. The resulting dataset supports semantic segmentation, instance segmentation, and image–point-cloud multimodal learning, providing a reliable data foundation for subsequent model training and evaluation.

## 3 Methods

We propose MDA-SegNet, a multimodal framework for individual rubber tree segmentation in structurally complex plantation scenes. As shown in Fig. 2, the framework consists of three main components: Multimodal Deformable Encoding (MDE), Z-order Selective Mamba (ZOS-Mamba), and Adaptive Lemming Optimization Clustering (ALOC). MDE introduces top-view crown-boundary information into 3D point-cloud feature learning to improve boundary discrimination in overlapping canopy regions. ZOS-Mamba models spatial continuity under uneven vertical point-density distributions, reducing feature discontinuity between dense crowns and sparse lower stems. ALOC further refines instance separation by adapting the clustering process to local structural variation. Together, these components form a boundary-aware, density-adaptive, and instance-oriented segmentation pipeline for individual rubber tree parsing.

### 3.1 Multimodal Deformable Encoding

In rubber plantation instance segmentation, point-cloud data mainly provide geometric information, while texture and boundary cues are relatively limited. When adjacent crowns overlap, points near crown-transition regions often show similar spatial distributions, which can lead to boundary confusion and unstable instance separation [38,39]. To address this problem, we propose the Multimodal Deformable Encoding (MDE) module for boundary-guided 2D–3D feature fusion in point-cloud instance segmentation. By introducing top-view crown-boundary cues that are consistent with the horizontal distribution of UAV LiDAR points, MDE strengthens the boundary representation of geometry-only point-cloud features while preserving the spatial structure of rubber tree crowns. The module uses a structured JSON file generated from nadir imagery as the 2D modality and integrates it with the geometric features of the 3D point cloud. In this study, the structured JSON file stores crown-related semantic and geometric information extracted from the nadir image, mainly including crown/background labels, crown boundary coordinates, region location information, and 2D–3D spatial alignment parameters. These metadata provide compact top-view boundary guidance for subsequent 2D–3D feature alignment and structural refinement in MDE. Unlike methods that rely only on geometric cues, MDE introduces explicit crown-boundary information from the top view to guide point-wise feature learning. In addition, deformable interpolation and global-dependency modeling are used to adapt the sampling process to local canopy structures and capture long-range associations within non-rigid crown regions, thereby improving boundary consistency and instance-level segmentation.

First, the structured JSON metadata are encoded by the JSON encoder *E*_JSON_ to generate a semantic vector *F*_*s*_. This vector represents the semantic distinction between crown and non-target regions in the nadir-view annotation and provides compact boundary-related information for the following structural encoding process. The semantic vector is then passed through a multilayer perceptron (MLP) to generate the structural template parameters:

(1) $$H_{s}=reshape\left({MLP}_{\text{hypercube}}\left(F_{s}\right),\left[8,D\right]\right)$$

Where $H_{s}\in R^{8\times D}$ represents the semantic template vertices of a hypercubic structure, which serves as the geometric control reference for subsequent trilinear interpolation, and *D* denotes the feature dimension of each template vertex. Based on this semantic structural template, a Minkowski U-Net is introduced after the JSON decoder *D*_JSON_ to perform sparse multi-scale feature encoding. Since the structural template is sparse in the 3D space, the Minkowski framework can extract multi-scale structural representations while avoiding redundant dense voxel convolution. This provides geometry-aware semantic guidance before interpolation-based fusion.

Meanwhile, the original 3D coordinates of the point cloud are normalized, and the trilinear interpolation weights corresponding to the eight template vertices are calculated using the normalized coordinates (*x*, *y*, *z*):

(2) $$w_{1}=\left(1-x\right)\left(1-y\right)\left(1-z\right),\;w_{2}=x\left(1-y\right)\left(1-z\right)$$

(3) $$w_{3}=\left(1-x\right)y\left(1-z\right),\;w_{4}=xy\left(1-z\right)$$

(4) $$w_{5}=\left(1-x\right)\left(1-y\right)z,\;w_{6}=x\left(1-y\right)z$$

(5) $$w_{7}=\left(1-x\right)yz,\;w_{8}=xyz$$

These weights are concatenated to form the interpolation weight matrix $W_{\text{interp}}\in R^{N\times 8}$, which encodes the relative geometric position of each point within the template space. The semantic structural template and the trilinear interpolation weights are then fused through a weighted summation to produce the aligned feature representation:

(6) $$F_{\text{fused}}\left[n,d\right]=\overset{8}{\underset{w=1}{\sum }}W_{\text{interp}}\left[n,w\right]\cdot H_{s}\left[w,d\right]$$

where *n* denotes the point index, *d* denotes the feature dimension, and *w* denotes the vertex index used for interpolation. Through this operation, the semantic information from the top-view annotation is embedded into the 3D structural modeling process, providing a boundary-enhanced feature basis for subsequent path selection, gating, and dynamic convolution.

Next, the local point-cloud features are combined with the semantic vector to form a unified representation for dynamic path selection. Soft weights are generated to determine the contribution of different convolutional branches for each point:

(7) $$B\left(F_{\text{fused}}\right)=softmax\left({MLP}_{\text{b}}\left(F_{\text{fused}}\right)\right)$$

where the softmax function is applied to the output of the MLP, and *F*_fused_ denotes the fused feature representation. This branch-selection mechanism allows the network to adaptively select suitable convolutional operations according to both semantic guidance and local geometric structure.

A sparse gating mechanism is then used to select semantically relevant points:

(8) $$G_{\text{mask}}=\sigma \left({MLP}_{\text{g}}\left(\left[P,expand\left(F_{s}\right)\right]\right)\right),\;P_{\text{masked}}=P\cdot 1\left(G_{\text{mask}}>\tau \right)$$

where MLP_g_ is used to compute the gating mask, *P* denotes the original 3D point cloud, expand(*F*_*s*_) aligns the semantic feature with the point-cloud size, *P*_masked_ denotes the point subset selected by sparse gating, *τ* is the gating threshold, and **1**(⋅) denotes the indicator function. This selection mechanism enables the module to focus on boundary- and crown-related regions while reducing redundant computation in less informative areas.

Although the above operations introduce semantic guidance into local geometric modeling, local feature interactions alone are insufficient to capture the global structure of overlapping crowns. Therefore, the fused features are further reorganized into multiple structural subspaces:

(9) $$Q_{\text{states}}=reshape\left(Q\left(x,p,F_{\text{json}}\right),\left[N,4,K\right]\right)$$

where *K* denotes the dimension of each subspace group, 4 denotes the number of subspaces, *N* denotes the number of points, and reshape(⋅) indicates tensor rearrangement. *F*_json_ represents the structured semantic feature input, and *p* denotes the spatial position of a point. An entanglement modeling operation is then applied to each structural subspace:

(10) $$Q_{\text{entangle},j}=\frac{1}{N}\overset{N}{\underset{i=1}{\sum }}Q_{\text{states}}\left[i,j,:\right]\cdot Q_{\text{states}}{\left[i,j,:\right]}^{T}$$

The resulting set ${\left\{Q_{\text{entangle},j}\right\}}_{j=1}^{4}$ represents the global dependency relationships among different structural subspaces. This operation strengthens the interaction between local point-wise features and the global topological structure, allowing boundary-related information to be propagated across overlapping crown regions.

Finally, dynamic convolution kernels are generated, and a structural residual connection is introduced:

(11) $$K_{\text{dyn}}={MLP}_{\text{kernel}}\left(P_{\text{masked}}\right),\;Y_{\text{out}}=W_{\text{skip}}\cdot P+mean\left(Q_{\text{entangle}}\right)\cdot K_{\text{dyn}}$$

where *K*_dyn_ denotes the dynamically generated convolution kernel, *W*_skip_ ⋅ *P* denotes the residual connection, and *Y*_out_ denotes the output feature after dynamic convolution. This design combines the original geometric pathway with the global interaction information, enabling semantically guided point-wise feature updating while preserving structural continuity.

Overall, MDE differs from simple feature concatenation or standard cross-attention-based fusion by embedding structured top-view crown cues into the 3D geometric modeling process. It first generates a semantic structural template from the JSON metadata, then aligns this template with 3D point coordinates through trilinear interpolation, and finally refines the fused representation through dynamic path selection, sparse gating, global dependency modeling, and dynamic convolution. In this way, MDE provides explicit boundary-aware guidance for separating adjacent rubber tree crowns while maintaining the spatial integrity of individual tree structures.

### 3.2 Z-order and Selective Mamba

To address the feature imbalance caused by uneven vertical point-density distributions in rubber tree point clouds, we propose the Z-order Selective Mamba (ZOS-Mamba) module. Rubber tree point clouds usually contain dense returns in the upper canopy and relatively sparse returns around the lower stems and branches. This density difference can weaken the continuity of point-wise feature representation and lead to incomplete crown–stem association during segmentation. ZOS-Mamba is designed to combine locality-preserving spatial serialization with selective state-space modeling, thereby capturing structural dependencies from dense canopy regions to sparse lower-stem regions and improving the stability of individual-tree segmentation.

Initially, the original 3D point cloud $P={\left\{\left(x_{i},y_{i},z_{i}\right)\right\}}_{i=1}^{N}$ is voxelized to construct a structured voxel representation:

(12) $$V_{x,y,z}=\left\{p|p\in P,\;and\;p\;within\;voxel\;\left(x,y,z\right)\right\}$$

Through voxelization, the irregular point distribution is converted into a structured spatial representation. Then, Z-order encoding is applied to the voxel coordinates. By interleaving the binary bits of the three-dimensional coordinates, a one-dimensional spatial index *Z*_index_ is generated:

(13) $$Z_{\text{index}}=\overset{b-1}{\underset{i=0}{\sum }}\left(\left(\left(x\&\left(1\ll i\right)\right)\ll \left(2i\right)\right)+\left(\left(y\&\left(1\ll i\right)\right)\ll \left(2i+1\right)\right)+\left(\left(z\&\left(1\ll i\right)\right)\ll \left(2i+2\right)\right)\right)$$

where *b* denotes the encoding precision, ≪ denotes the bitwise left-shift operation, and *&* denotes the bitwise AND operation. This Z-order mapping converts the 3D voxelized point cloud into a one-dimensional sequence while preserving local spatial adjacency. In this way, nearby points in the 3D space tend to remain close in the serialized sequence, which provides a structured input for subsequent sequential modeling.

The serialized feature sequence is then processed by Selective Mamba. Conventional Mamba is based on a state-space model (SSM), where the hidden state and output are updated as follows:

(14) $$x_{t+1}=e^{\Delta_{t}A}x_{t}+\Delta_{t}B_{t}u_{t},\;y_{t}=C_{t}x_{t}+Du_{t}$$

where *u*_*t*_ denotes the input feature at step *t*, *x*_*t*_ denotes the hidden state, and *y*_*t*_ denotes the output feature. The term $e^{\Delta_{t}A}$ is obtained from the discretization of the continuous state transition matrix. In this formulation, *A* and *D* are static parameters, whereas Δ_*t*_, *B*_*t*_, and *C*_*t*_ are input-dependent parameters. These dynamic parameters are generated through linear projection:

(15) $$\left(\Delta_{t},B_{t},C_{t}\right)=LinearProjection\left(u_{t}\right),\;\Delta_{t}=Softplus\left(\Delta_{t}\right)=\ln\left(1+e^{\Delta_{t}}\right)$$

To improve computational efficiency, the selective scan operation is implemented in a parallel form:

(16) $$X=cumsum\left(e^{\Delta A}⊙\Delta B⊙u\right),\;y=CX+Du$$

where ⊙ denotes element-wise multiplication, and cumsum(⋅) denotes cumulative summation along the sequence dimension. This parallel formulation preserves the sequential dependency modeling of Mamba while improving computational efficiency during feature propagation.

After the Selective Mamba operation, RMSNorm is applied to stabilize the feature distribution:

(17) $$y_{\text{norm}}=\frac{y}{\sqrt{\frac{1}{d}\sum_{i=1}^{d}y_{i}^{2}+\epsilon }}⊙w$$

where *d* denotes the feature dimension, *ϵ* is a small constant used to avoid division by zero, and *w* is a trainable scaling parameter. RMSNorm reduces scale variation among feature dimensions, which helps stabilize optimization and improve the generalization of the Mamba-processed features.

Finally, an inverse Z-order mapping is used to restore the processed sequence features to their original 3D spatial order. Let $Z_{\text{index}\;}^{-1}$ denote the inverse mapping from the serialized order to the original point order. The restored feature representation is expressed as:

(18) $$y_{\text{restored}}=y_{\text{norm}}\left[Z_{\text{index}}^{-1}\right]$$

This restoration step ensures that the features processed by Selective Mamba can be mapped back to their corresponding spatial positions in the original point cloud. Therefore, subsequent point-cloud operations can be performed while maintaining spatial consistency.

In summary, ZOS-Mamba addresses the vertical density imbalance of rubber tree point clouds by combining Z-order spatial serialization with selective state-space modeling. Z-order encoding preserves local spatial adjacency during sequence construction, while Selective Mamba captures long-range structural dependencies along the serialized point sequence. This design helps maintain feature continuity between dense upper-canopy regions and sparse lower-stem regions, reducing feature fragmentation caused by uneven point density and providing a more stable structural representation for individual rubber tree segmentation.

### 3.3 Adaptive Lemming Optimization Clustering

Rubber trees at different growth stages usually exhibit substantial variation in crown size, branching pattern, stem visibility, and canopy closure. Such structural heterogeneity increases the difficulty of instance-level crown separation, especially in areas with heavy crown overlap or irregular canopy morphology. To improve clustering adaptability under these conditions, we develop an Adaptive Lemming Optimization Clustering (ALOC) module. ALOC adjusts the clustering process according to the structural characteristics of the point cloud and dynamically balances global exploration and local refinement during optimization. This design improves the robustness of instance separation under variable canopy structures and complex plantation conditions.

First, ALOC adopts an input-size-aware strategy to select the clustering process according to the scale and complexity of the point-cloud segment. For segments with fewer points, the K-Nearest Neighbor (KNN) algorithm is used to improve computational efficiency. For larger or structurally more complex segments, Mean Shift is used to capture the cluster distribution more robustly.

During the early optimization stage, Lévy flight is introduced to increase the global search ability and reduce the risk of premature convergence to local optima. The corresponding step length is defined as:

(19) $$step=\frac{u}{{\left|v\right|}^{1/\beta }},\;u∼N\left(0,\sigma^{2}\right),\;v∼N\left(0,1\right),\;\beta =1.5$$

where

(20) $$\sigma ={\left(\frac{\Gamma \left(1+\beta \right)\sin\left(\pi \beta /2\right)}{\Gamma \left(\left(1+\beta \right)/2\right)\beta 2^{\left(\beta -1\right)/2}}\right)}^{1/\beta }$$

Here, *u* is sampled from a normal distribution $N\left(0,\sigma^{2}\right)$, *v* is sampled from a standard normal distribution N(0,1), *β* is the Lévy distribution parameter and is set to 1.5 in this study, *σ* is the scale parameter, and Γ(⋅) denotes the Gamma function.

The search range is further adjusted using a dynamic control function related to the training progress:

(21) $$E=2\cdot \ln\left(1/random\left(\right)\right)\cdot \theta ,\;\theta =2\cdot \arctan\left(1-\frac{iteration}{max\text{\_}iter}\right)$$

where *E* denotes the dynamic control factor for the search range, random() denotes a uniformly distributed random value in 01, *θ* is the angular control parameter that changes with the number of iterations, iteration is the current iteration number, and max_iter is the maximum number of iterations. When *E* ≤ 1, the optimization process gradually shifts from global exploration to local exploitation. At this stage, ALOC updates the clustering parameters using the DS and GFT mechanisms:

(22) $$\theta^{\text{new}}=\theta^{\text{best}}+F\cdot RB\cdot \left(r_{1}\cdot \left(\theta^{\text{best}}-\theta^{\text{current}}\right)\right)$$

(23) $$\theta^{\text{new}}=\theta^{\text{current}}+F\cdot r_{2}\cdot \left(\theta^{\text{best}}-\theta^{\text{current}}\right)$$

where *F* denotes the scaling factor, and *R*, *B*, *r*_1_, and *r*_2_ denote perturbation terms generated by dynamic sampling. These terms control the step length and update direction of the clustering parameters. This update strategy accelerates convergence when the optimization process becomes stable while maintaining the adaptability of instance separation.

To further reduce the possibility of being trapped in local optima caused by repetitive canopy structures, a spiral search strategy is introduced for local refinement. The parameter update is defined as:

(24) $$\theta^{\text{new}}=\theta^{\text{best}}+F\cdot \theta^{\text{current}}\cdot spiral\cdot random\left(\right)$$

(25) $$spiral=radius\cdot \left(\sin\left(2\pi r_{3}\right)+\cos\left(2\pi r_{3}\right)\right),\;radius=\sqrt{\sum {\left(\theta^{\text{best}}-\theta^{\text{current}}\right)}^{2}}$$

where spiral denotes the spiral perturbation factor, *r*_3_ is a random variable, and radius represents the distance between the current parameter state and the best parameter state. This strategy combines directional guidance, adaptive amplitude adjustment, and stochastic perturbation, thereby improving local refinement while preserving the ability to escape local optima.

Whether the updated parameters are accepted is determined by a feedback score from the semantic evaluation module:

(26) $${score}_{\text{eval}}=\alpha \cdot {mean}_{f_{1}}+\left(1-\alpha \right)\cdot {max}_{f_{1}},\;\alpha =0.7$$

where score_eval_ denotes the feedback score, *α* is the weight coefficient, ${mean}_{F_{1}}$ denotes the average F-score over all validation samples, and $\max_{F_{1}}$ denotes the maximum F-score among validation samples. The parameter update is accepted only when score_eval_ exceeds the historical best score. This feedback mechanism reduces unstable updates caused by local noise or excessive perturbation and supports stable optimization.

Overall, ALOC forms a closed-loop adaptive clustering mechanism that coordinates feature evaluation and clustering-parameter adjustment. By dynamically switching between global exploration and local refinement, ALOC improves instance separation under heterogeneous canopy structures, variable crown scales, and overlapping rubber tree crowns. This module therefore contributes to more stable individual-tree segmentation in structurally complex rubber plantation scenes.

## 4 Experiments

To comprehensively validate the effectiveness, robustness, and practical applicability of the proposed MDA-SegNet for individual rubber tree segmentation, extensive experiments were conducted on the constructed UAV LiDAR dataset. This section systematically details the experimental process and results.

### 4.1 Experimental environment and parameter setting

All experiments were conducted on a computing platform equipped with an Intel Xeon CPU, an NVIDIA RTX 3090 GPU (24 GB), and 43.0 GB RAM, running Ubuntu 18.04. The implementation was based on Python 3.8 and CUDA 11.1. The model was trained using the Adam optimizer for 150 epochs with a batch size of 16. The detailed hardware/software environment and training settings are summarized in Table 1.

**Table 1:** Experimental settings and computational efficiency of MDA-SegNet.

Category	Item	Value
Hardware/software environment	CPU	12 vCPU Intel Xeon Platinum 8255C @ 2.50 GHz
	GPU	NVIDIA RTX 3090 24 GB
	RAM	43.0 GB
	Operating system	Ubuntu 18.04
	Python version	Python 3.8
	CUDA version	CUDA 11.1
Training settings	Optimizer	Adam
	Epochs	150
	Batch size	16
Computational cost and efficiency	Trainable parameters	11.911 M
	FLOPs	46.6 GFLOPs
	Peak GPU memory	0.52 GB
	Training time/epoch	3.80 min
	Inference time/sample	43.29 ms

### 4.2 Computational complexity and efficiency analysis

To further evaluate the computational cost and efficiency of the proposed MDA-SegNet, the number of trainable parameters, floating-point operations, peak GPU memory consumption, training time, and inference time were recorded under the same experimental environment. The computational complexity and efficiency results are summarized in Table 1.

As shown in Table 1, the proposed MDA-SegNet contains 11.911M trainable parameters and requires 46.6 GFLOPs for a single forward pass. The peak GPU memory consumption is 0.52 GB. In terms of computational efficiency, the average training time is 3.80 min per epoch, and the average inference time is 43.29 ms per sample. These results provide a quantitative reference for evaluating the computational cost and training/inference efficiency of the proposed framework under the current experimental setting.

### 4.3 Evaluation metrics

To comprehensively evaluate the performance of the proposed model for individual rubber tree crown segmentation, both semantic-level and instance-level metrics are adopted, including mIoU, F-score, AP, Precision (Pre), and Recall (Rec).

First, the mean Intersection over Union (mIoU) is used as a semantic-level metric to measure the average spatial overlap between the predicted segmentation and the ground truth across all semantic classes. It is defined as

(27) $$mIoU=\frac{1}{N}\overset{N}{\underset{i=1}{\sum }}\frac{TP_{i}}{TP_{i}+FP_{i}+FN_{i}}$$

where *N* denotes the number of semantic classes. In this study, *N* = 2, corresponding to background and crown. *TP*_*i*_, *FP*_*i*_, and *FN*_*i*_ represent the numbers of true positive, false positive, and false negative point-level samples of class *i*, respectively.

In addition to semantic overlap quality, instance-level metrics are further used to evaluate the delineation performance of individual tree crowns. Specifically, a predicted crown instance is regarded as a true positive (TP) if it can be matched to a ground-truth crown instance under a predefined IoU threshold *τ*. Unmatched predicted instances are counted as false positives (FP), whereas unmatched ground-truth instances are counted as false negatives (FN). Based on this instance-level matching strategy, Precision and Recall are defined as:

(28) $$Pre=\frac{TP}{TP+FP}$$

(29) $$Rec=\frac{TP}{TP+FN}$$

where Pre denotes the proportion of correctly predicted crown instances among all predicted instances, and Rec denotes the proportion of correctly identified ground-truth crown instances.

To further assess the balance between precision and recall, the F-score is calculated as:

(30) $$F-score=\frac{2\cdot Pre\cdot Rec}{Pre+Rec}$$

Moreover, for a given IoU threshold *τ*, the Average Precision (AP) is computed as the area under the Precision-Recall curve:

(31) $$AP_{\tau }=\int_{0}^{1}p\left(r\right)\;dr$$

where *p*(*r*) denotes precision as a function of recall *r*.

By combining the semantic-level metric mIoU with the instance-level metrics Pre, Rec, F-score, and AP, the segmentation results can be comprehensively evaluated from the perspectives of both regional overlap quality and individual crown matching performance.

### 4.4 Module effectiveness experiment

To quantitatively evaluate the effectiveness and individual role of the proposed components, we conducted a series of module effectiveness experiments for MDE, ZOS-Mamba, and ALOC. As summarized in Table 2, each module was assessed under its corresponding experimental setting while keeping the remaining training and evaluation protocol consistent. These experiments were designed to clarify how each proposed component contributes to semantic overlap quality and instance-level crown delineation.

**Table 2:** Quantitative effectiveness evaluation of the proposed MDE, ZOS-Mamba, and ALOC modules.

Types	Experimental setting	Evaluation Metrics (%)				
		mIoU	F-score	AP	Pre	Rec
MDE	single-modal 3D-only	60.59	78.98	64.88	88.95	71.02
	Front View	62.34	78.74	63.24	81.02	76.58
	Left View	67.28	80.65	66.06	85.16	76.59
	Right View	65.37	80.79	66.13	84.30	77.55
	**Top View**	**72.16**	**84.77**	**73.27**	**90.70**	**79.56**
ZOS-Mamba	KNN	65.25	80.41	65.28	83.62	77.43
	PointConv	63.19	79.62	64.43	86.64	73.64
	KPConv	66.07	81.30	67.43	87.39	76.00
	CNN	65.20	82.88	70.30	86.57	79.49
	Transformer	69.33	83.68	70.73	87.91	79.84
	**ZOS-Mamba**	**71.94**	**84.32**	**71.68**	**89.27**	**79.89**
ALOC	MeanShift	64.17	76.39	59.61	84.32	69.82
	DPC	67.46	73.90	56.09	89.76	62.81
	DBSCAN	66.83	79.67	64.61	86.05	74.11
	**ALOC**	**72.09**	**83.30**	**70.18**	**90.30**	**77.31**

#### 4.4.1 Effectiveness experiment of MDE

To evaluate the contribution of MDE, the multimodal fusion component of MDA-SegNet, we conducted effectiveness experiments by varying the structured input configuration while keeping the remaining model architecture and training protocol unchanged. MDE is designed to introduce structured 2D crown-boundary cues into 3D UAV LiDAR-based individual rubber tree crown segmentation, thereby compensating for the limited boundary separability of geometry-only point-cloud representations through 2D–3D feature alignment.

A single-modal 3D-only setting was first included as a reference to quantify the performance obtained using LiDAR geometry alone. In addition, JSON-based structured inputs generated from the front, left, right, and top views were compared to investigate how different 2D boundary cues affect point-cloud crown segmentation.

As shown in the MDE section of Table 2, the top-view JSON input achieved the best performance among all structured input configurations. Compared with the single-modal 3D-only setting, it improved mIoU, F-score, AP, and Recall by 11.57, 5.79, 8.39, and 8.54 percentage points, respectively, while also yielding a slightly higher Precision. These improvements demonstrate that the structured top-view boundary cues provide complementary information beyond LiDAR geometry alone.

Compared with front-, left-, and right-view inputs, the top-view input provides a more consistent representation of crown contours and horizontal canopy extent, which is particularly important for separating adjacent rubber tree crowns under overlapping conditions. In contrast, non-top-view inputs are more likely to be affected by crown occlusion and projection distortion, resulting in less stable boundary guidance. Therefore, MDE mainly contributes to the framework by strengthening boundary-aware 2D–3D structural alignment and improving the completeness of individual crown delineation.

#### 4.4.2 Effectiveness experiment of ZOS-Mamba

To evaluate the structural modeling effectiveness of the proposed ZOS-Mamba module, we conducted a set of module replacement experiments while keeping the remaining model architecture, input configuration, and training protocol unchanged. Specifically, ZOS-Mamba was replaced with several representative feature modeling strategies, including KNN-based local neighborhood modeling [40], PointConv [41], KPConv [42], a CNN-based module [43], and a Transformer-based module [44]. These settings were used to assess the influence of different spatial feature modeling mechanisms on individual rubber tree crown segmentation. Rubber tree point clouds usually exhibit dense upper-canopy returns and sparse lower-trunk returns, making vertical structural continuity difficult to preserve during feature modeling.

As shown in the ZOS-Mamba section of Table 2, ZOS-Mamba achieved the best overall performance among all replacement settings. Compared with the CNN-based setting, it increased mIoU by 6.74 percentage points, indicating improved semantic-level structural consistency. Compared with the Transformer-based setting, ZOS-Mamba further improved mIoU by 2.61 percentage points and achieved higher AP and Precision, while maintaining a comparable Recall. In addition, ZOS-Mamba consistently outperformed the KNN-, PointConv-, and KPConv-based settings across the main evaluation metrics.

These results indicate that ZOS-Mamba provides a more effective representation for rubber tree point clouds with pronounced canopy-to-trunk density variation. The improvements suggest that its spatially ordered structural modeling is beneficial for maintaining vertical structural continuity and improving density-aware feature representation. Therefore, ZOS-Mamba mainly contributes to the framework by enhancing semantic overlap quality and instance-level reliability under density-varying point-cloud conditions.

#### 4.4.3 Effectiveness experiment of ALOC

To evaluate the contribution of ALOC to instance-level clustering under structural heterogeneity, we conducted module replacement experiments while keeping the remaining model architecture, input configuration, and training protocol unchanged. Specifically, ALOC was replaced with three representative clustering strategies, including MeanShift [45], Dynamic Prototype Clustering (DPC) [46], and DBSCAN [47]. These settings were used to assess the influence of different clustering mechanisms on individual crown separation under variable canopy sizes, branching patterns, and growth-stage differences.

As shown in the ALOC section of Table 2, ALOC achieved the best overall performance among all clustering settings, with 72.09% mIoU, 83.30% F-score, 70.18% AP, 90.30% Precision, and 77.31% Recall. Compared with DBSCAN, ALOC improved mIoU, F-score, AP, and Recall by 5.26, 3.63, 5.57, and 3.20 percentage points, respectively. Compared with DPC, although DPC achieved relatively high Precision, its Recall and AP were much lower, indicating that it tended to preserve high-confidence clusters while missing incomplete or irregular crown instances. In contrast, ALOC achieved a better balance between Precision and Recall, leading to improved F-score and AP.

These results indicate that ALOC provides more stable instance separation under heterogeneous crown structures. MeanShift is limited by its fixed bandwidth, while DBSCAN depends strongly on predefined neighborhood radius and density thresholds. These static clustering assumptions are less suitable for rubber tree crowns with varying canopy scales and irregular branching morphology. By contrast, ALOC contributes to the framework by improving adaptive clustering stability, preserving instance completeness, and enhancing crown separation performance under multi-stage and structurally heterogeneous plantation conditions.

### 4.5 Ablation experiments

To examine the contribution and compatibility of the three core modules in the proposed framework, we conducted a series of module-based ablation experiments. MDE, ZOS-Mamba, and ALOC were individually integrated into the baseline model, and different module combinations were further evaluated to analyze their joint effects. The quantitative results are presented in Table 3.

**Table 3:** Quantitative results of the ablation experiments for individual modules.

Methods			Evaluation Metrics (%)				
MDE	ZOS-Mamba	ALOC	mIoU	F-score	AP	Pre	Rec
			66.41	80.46	66.82	85.91	75.66
*✓*			72.16	84.77	73.27	90.70	79.56
	*✓*		71.94	84.32	71.68	89.27	79.89
		*✓*	72.09	83.30	70.18	90.30	77.31
*✓*	*✓*		75.83	85.59	72.95	90.12	81.49
	*✓*	*✓*	75.60	85.31	73.78	91.30	80.06
*✓*		*✓*	76.46	85.81	73.13	91.41	80.85
*✓*	*✓*	*✓*	**76.58**	**87.32**	**76.39**	**92.23**	**82.90**

**Fig. 3:**
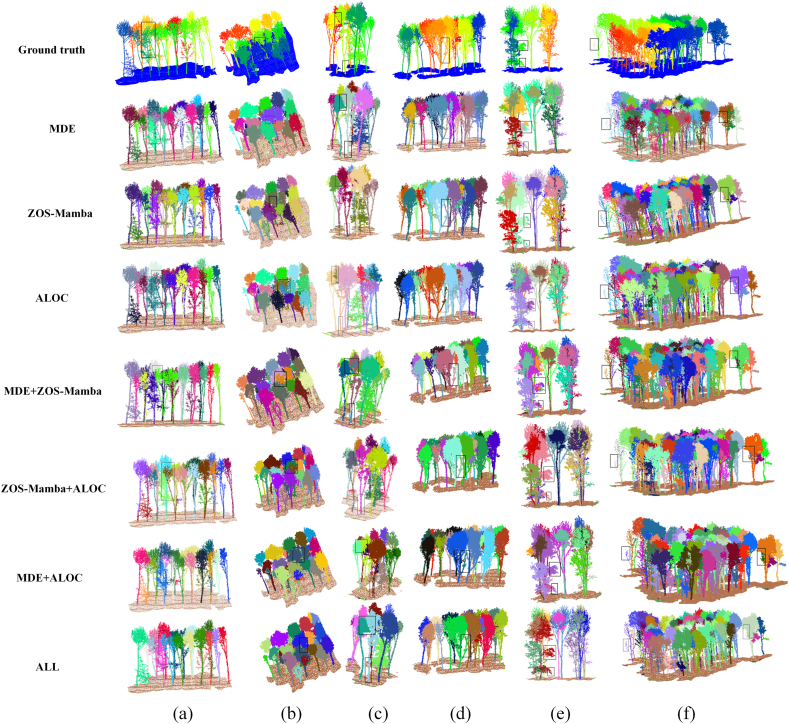
Qualitative results of ablation experiments on six challenging forest scenes (a–f), evaluating the effects of MDE, ZOS-Mamba, and ALOC modules. Each row corresponds to a specific module configuration, from the baseline to ALL. The figure highlights differences in segmentation performance under high-density, vertically layered, and phenologically variable conditions.

As shown in Table 3, the baseline model without the three proposed modules achieved 66.41% mIoU, 80.46% F-score, and 66.82% AP. After introducing MDE alone, the model improved to 72.16% mIoU, 84.77% F-score, and 73.27% AP. The increases of 5.75 percentage points in mIoU and 6.45 percentage points in AP indicate that the top-view boundary information provides useful complementary guidance for separating adjacent crowns. This improvement is also reflected in Fig. 3(a) and (b), where MDE reduces boundary confusion in high-density canopy regions.

With only ZOS-Mamba added, the model achieved 71.94% mIoU and 84.32% F-score, showing a clear improvement over the baseline. Recall also increased from 75.66% to 79.89%, suggesting that ZOS-Mamba helps preserve more complete tree instances. This result is consistent with its role in modeling spatially ordered structural features under uneven point-density distributions. As shown in Fig. 3(c) and (d), the module helps maintain the structural continuity between dense canopy regions and sparse lower-stem regions.

When ALOC was introduced alone, the model achieved 72.09% mIoU and 83.30% F-score. Compared with the baseline, Precision increased from 85.91% to 90.30%, indicating that ALOC improves the reliability of instance assignment during crown separation. This suggests that adaptive clustering is beneficial for handling rubber trees with different crown sizes, branching patterns, and growth stages. The qualitative results in Fig. 3(e) and (f) further show clearer instance boundaries in structurally heterogeneous canopy regions.

The combined-module results further demonstrate the complementarity among the three components. The combination of MDE and ZOS-Mamba achieved 75.83% mIoU and 85.59% F-score, while the combination of MDE and ALOC achieved 76.46% mIoU and 85.81% F-score. These results suggest that boundary-aware multimodal fusion, vertical structural modeling, and adaptive clustering improve the segmentation performance from different aspects.

When all three modules were integrated, the model achieved the best overall performance, with 76.58% mIoU, 87.32% F-score, 76.39% AP, 92.23% Precision, and 82.90% Recall. Compared with the baseline, the complete model improved mIoU, F-score, and AP by 10.17, 6.86, and 9.57 percentage points, respectively. These results indicate that MDE, ZOS-Mamba, and ALOC play complementary roles in crown-boundary discrimination, vertical structural continuity, and adaptive instance clustering, leading to more stable individual-tree segmentation in dense rubber plantation scenes.

### 4.6 Comparison on RT-set

**Fig. 4:**
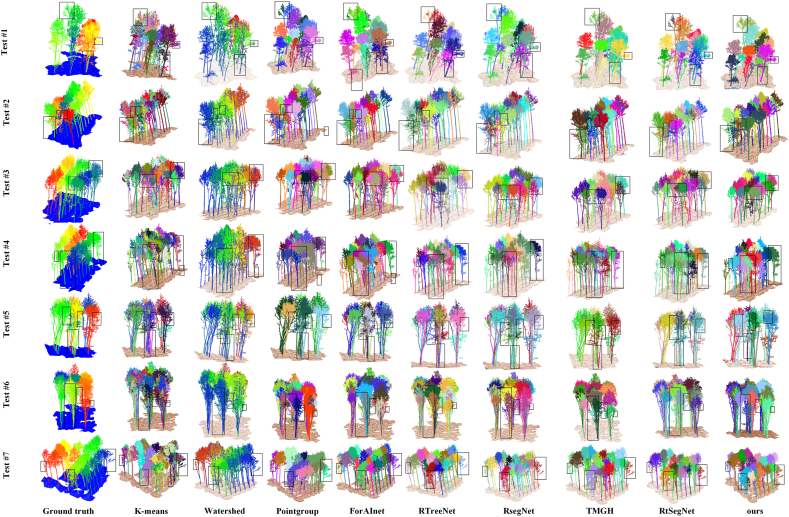
Qualitative comparison of individual tree segmentation results across seven representative test plots (Test#1–Test#7) under different ecological conditions. From left to right: ground truth, K-means, Watershed, PointGroup, ForAInet, RTreeNet, RsegNet and the proposed method (ours). Black boxes highlight typical regions of crown overlap, structural discontinuity, and developmental heterogeneity.

To evaluate the segmentation performance of the proposed MDA-SegNet on RT-Set, we compared it with eight representative baseline methods, covering conventional clustering or rule-based approaches, general point-cloud instance segmentation frameworks, forest-oriented LiDAR models, and recent tree- or rubber-tree-specific segmentation networks. Specifically, the compared methods include K-means [48], Watershed [49], PointGroup [50], ForAInet [51], RTreeNet, RsegNet, TMGH [52], and RtSegNet. To ensure a fair comparison, all baseline models were evaluated on RT-Set under the same experimental protocol. Quantitative results are summarized in Table 4, and qualitative segmentation results on seven representative test blocks are shown in Fig. 4.

**Table 4:** Instance segmentation results on RT-Set.

Methods	Evaluation Metrics (%)				
	mIoU	F-score	AP	Pre	Rec
K-means	30.19	37.96	—	48.79	31.06
Watershed	39.04	46.84	40.73	48.86	44.98
PointGroup	63.47	66.11	61.28	69.25	63.24
ForAInet	65.81	82.27	71.43	86.27	78.62
RTreeNet	74.35	84.76	72.76	89.96	80.92
RsegNet	75.72	85.81	73.96	91.27	81.60
TMGH	73.54	79.12	71.68	85.49	73.64
RtSegNet	76.16	86.80	75.60	91.96	82.18
**Ours**	**76.58**	**87.32**	**76.39**	**92.23**	**82.90**

As shown in Table 4, MDA-SegNet achieved the best overall performance among all compared methods, with 76.58% mIoU, 87.32% F-score, 76.39% AP, 92.23% Precision, and 82.90% Recall. Compared with the recent RtSegNet method, MDA-SegNet improved mIoU, F-score, AP, Precision, and Recall by 0.42, 0.52, 0.79, 0.27, and 0.72 percentage points, respectively. Although the numerical margins over RtSegNet are moderate, the proposed method consistently achieves the highest values across all five evaluation metrics, indicating a more balanced performance in both semantic overlap quality and instance-level crown delineation.

Compared with RsegNet, MDA-SegNet improved mIoU and AP by 0.86 and 2.43 percentage points, respectively. The larger improvement in AP indicates that the proposed framework provides better instance-level crown matching, especially under crown-overlap conditions. Compared with PointGroup and ForAInet, MDA-SegNet shows a larger performance advantage. PointGroup is a general point-cloud instance segmentation framework, while ForAInet is designed for forest inventory from LiDAR point clouds; however, both methods still show lower performance on RT-Set than the proposed method. This result suggests that existing general or forest-oriented point-cloud models may not fully adapt to the specific structural characteristics of dense rubber plantations, including adjacent crown interlacing and uneven canopy-to-trunk point density.

The qualitative results in Fig. 4 further illustrate the differences among the compared methods under different structural conditions. In Test #1–3, adjacent crowns are densely interlaced, and several baseline methods tend to produce merged crowns or unstable boundaries. By incorporating top-view boundary cues through MDE, MDA-SegNet provides clearer separation between neighboring rubber tree crowns. In Test #4–5, the point density changes substantially from the upper canopy to the lower stem region, and some methods show fragmented trunk–crown associations. The ZOS-Mamba module helps preserve vertical structural continuity and reduces this type of discontinuity. In Test #6–7, tree size and crown morphology vary across growth stages, and ALOC improves adaptive instance separation, leading to more stable crown boundaries.

Overall, the comparison on RT-Set demonstrates that MDA-SegNet achieves the best performance among conventional methods, general point-cloud segmentation frameworks, forest-oriented LiDAR models, and recent tree- or rubber-tree-specific segmentation baselines. The results indicate that the proposed multimodal boundary fusion, vertical structural modeling, and adaptive clustering strategy provide complementary benefits for individual rubber tree segmentation under dense canopy overlap, uneven vertical density, and heterogeneous growth-stage conditions.

### 4.7 Generalization and external robustness performance

To evaluate the generalization performance of the proposed method under heterogeneous forest point-cloud conditions, experiments were conducted on two public forest point-cloud datasets, namely FOR-instance and NIBIO_MLS. FOR-instance is a UAV laser scanning dataset collected from five forest regions, including Norway, the Czech Republic, Austria, New Zealand, and Australia. The test data contain 334 trees, with an average tree density of 425 trees ha^−1^, an average tree height of 21.6 m, and an average point density of 6747 pt m^−2^. The dataset covers diverse forest types, including boreal coniferous forest, temperate coniferous forest, temperate mixed deciduous forest, coniferous plantation forest, and dry sclerophyll forest. NIBIO_MLS is a mobile laser scanning dataset collected using a GeoSLAM ZEB-HORIZON scanner in managed boreal coniferous forests. The test data contain 258 trees, with an average tree density of 1467 trees ha^−1^, an average tree height of 14.7 m, and an average point density of 20,000 pt m^−2^. Therefore, these two datasets provide complementary testing conditions in terms of scanning platform, point density, forest type, and stand structure.

As shown in Table 5, the proposed method achieves competitive segmentation performance across the five FOR-instance subsets and the NIBIO_MLS dataset. On the FOR-instance UAV LiDAR benchmark, the proposed method obtains F-scores of 92.62%, 84.02%, 69.47%, 84.01%, and 64.85% on CULS, NIBIO, TUWIEN, SCION, and RMIT, respectively. Among these subsets, CULS, NIBIO, and SCION show relatively high segmentation accuracy, indicating that the proposed method can effectively capture individual-tree structures in coniferous and plantation forest scenes. For the more structurally complex TUWIEN and RMIT subsets, where mixed deciduous crowns, irregular branching patterns, and heterogeneous canopy structures make instance separation more challenging, the proposed method still maintains stable performance, with F-scores of 69.47% and 64.85%, respectively.

**Table 5:** Comparison of segmentation performance on public datasets.

Methods	Test dataset	Evaluation Metrics (%)				
		mIoU	F-score	AP	Pre	Rec
K-means	FOR-instance CULS	45.89	39.73	—	46.53	34.66
	FOR-instance NIBIO	35.10	34.16	—	30.37	39.04
	FOR-instance TUWIEN	20.67	30.79	—	39.60	25.19
	FOR-instance SCION	25.03	24.92	—	19.23	35.41
	FOR-instance RMIT	40.82	42.09	—	47.85	37.57
	NIBIO_MLS	21.80	19.92	—	19.51	20.35
PointGroup	FOR-instance CULS	66.85	76.92	50.59	80.37	73.75
	FOR-instance NIBIO	47.36	51.35	22.89	59.46	45.19
	FOR-instance TUWIEN	45.14	55.20	26.13	58.47	52.28
	FOR-instance SCION	41.71	59.81	11.96	61.43	58.27
	FOR-instance RMIT	38.62	49.16	15.19	59.67	41.80
	NIBIO_MLS	53.18	59.67	32.71	67.84	53.25
ForAInet	FOR-instance CULS	70.97	89.18	67.64	92.98	85.67
	FOR-instance NIBIO	69.29	77.05	53.86	87.49	68.84
	FOR-instance TUWIEN	66.24	66.76	40.83	74.08	60.75
	FOR-instance SCION	69.18	74.28	51.69	64.21	**88.10**
	FOR-instance RMIT	61.77	**65.65**	47.37	**69.25**	62.41
	NIBIO_MLS	63.09	67.80	50.60	76.05	61.17
RsegNet	FOR-instance CULS	75.84	**94.60**	70.21	**95.67**	**93.53**
	FOR-instance NIBIO	**73.26**	82.80	54.50	82.16	**83.46**
	FOR-instance TUWIEN	**66.47**	67.84	44.61	70.69	65.21
	FOR-instance SCION	70.68	81.28	51.39	75.56	87.93
	FOR-instance RMIT	**61.90**	64.89	40.06	61.34	**68.87**
	NIBIO_MLS	66.45	71.79	57.62	**80.94**	64.50
Ours	FOR-instance CULS	**76.38**	92.62	**70.70**	93.69	91.57
	FOR-instance NIBIO	72.49	**84.02**	**56.81**	**84.67**	83.38
	FOR-instance TUWIEN	65.72	**69.47**	**46.19**	**72.92**	**66.33**
	FOR-instance SCION	**70.95**	**84.01**	**53.64**	**84.45**	83.57
	FOR-instance RMIT	61.56	64.85	**43.88**	65.39	64.32
	NIBIO_MLS	**67.84**	**73.27**	**60.29**	80.07	**67.53**

Compared with the baseline methods, the proposed method shows a good balance between segmentation completeness and instance-level discrimination. On the FOR-instance NIBIO subset, the proposed method achieved an F-score of 84.02% and an AP of 56.81%, outperforming RsegNet by 1.22 and 2.31 percentage points, respectively. On the TUWIEN subset, where mixed deciduous crowns and irregular branching patterns increase the difficulty of instance separation, the proposed method improved the F-score from 67.84% to 69.47% and the AP from 44.61% to 46.19%. On the SCION subset, the proposed method also achieved higher F-score and AP than RsegNet, indicating better instance-level crown delineation in dense plantation forest scenes. These results suggest that the proposed model can maintain competitive generalization performance across UAV-based LiDAR datasets with different forest types and canopy structures.

The results on NIBIO_MLS further evaluate the cross-platform robustness of the proposed method. Unlike the UAV-based FOR-instance dataset, NIBIO_MLS was acquired using mobile laser scanning and has higher point density and different scanning geometry. Under this MLS setting, the proposed method achieved 67.84% mIoU, 73.27% F-score, and 60.29% AP, outperforming RsegNet by 1.39, 1.48, and 2.67 percentage points, respectively. This result indicates that the learned structural representation can be transferred from UAV LiDAR scenes to dense MLS forest point clouds, although performance differences across datasets still reflect the influence of scanning geometry and forest structure.

Overall, the results on FOR-instance and NIBIO_MLS demonstrate that the proposed method maintains competitive external robustness across public forest point-cloud datasets with different acquisition platforms, point densities, and stand structures. The relatively stable F-score and AP on several external datasets suggest that MDA-SegNet can distinguish individual tree instances under diverse canopy conditions, while highly irregular forest structures and cross-platform density variations remain challenging cases for further improvement.

## 5 Structural parameter extraction study

**Fig. 5:**
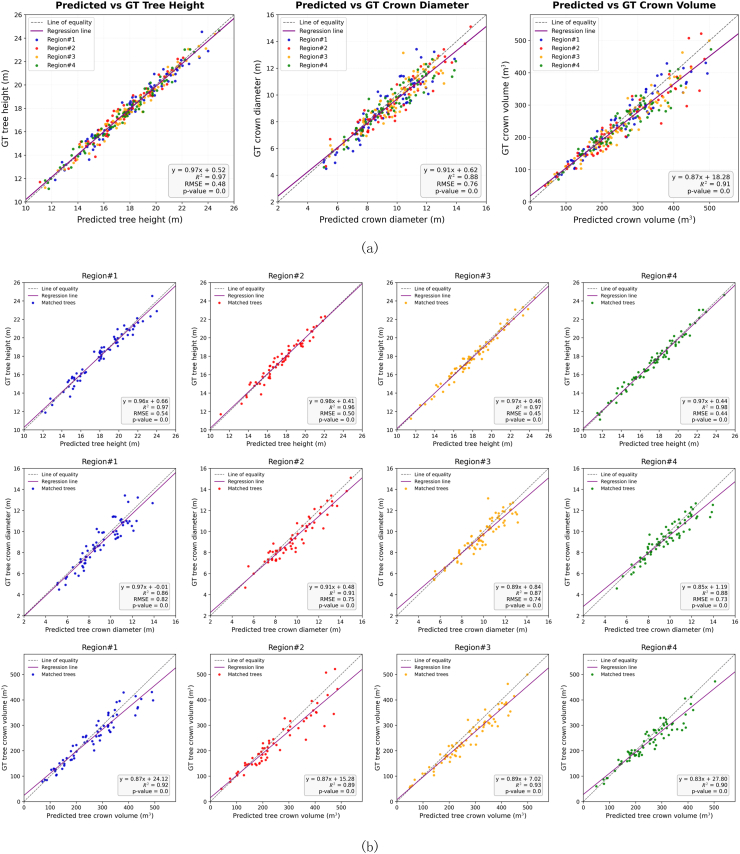
Scatter plots comparing the predicted structural parameters of rubber trees with ground truth (GT) measurements. (a) Overall estimation performance for tree height, crown diameter, and crown volume across all sampled trees. (b) Regional breakdown of the estimation results across the four individual forest blocks.

Four rubber plantation blocks in Danzhou, Hainan Province, China, containing approximately 270 individual rubber trees, were selected to evaluate the structural parameter extraction performance of the proposed framework. Three tree-level structural parameters, including tree height, crown diameter, and crown volume, were compared with ground-truth measurements. Fig. 5(a) shows the scatter plots between the predicted values and the ground-truth measurements. Tree height estimation achieved the strongest agreement, with an *R*^2^ of 0.97 and an RMSE of 0.48 m. For crown diameter and crown volume, which are more sensitive to crown boundary delineation and three-dimensional canopy reconstruction, the predicted values also showed strong correlations with the ground truth, with *R*^2^ values of 0.88 and 0.91, respectively. These results indicate that the proposed segmentation framework can provide reliable structural boundaries for subsequent tree-level parameter extraction.

Fig. 5(b) further presents the estimation results across the four individual plantation blocks. Tree height estimation remained stable among different blocks, while crown diameter and crown volume showed slight regional variation, with block-level *R*^2^ values ranging from 0.85 to 0.91 and from 0.89 to 0.93, respectively. These variations may be related to differences in crown overlap, local branching patterns, and growth conditions among the blocks. Nevertheless, the overall estimation accuracy remained consistent across different spatial subsets, suggesting that MDA-SegNet can maintain stable structural parameter extraction performance under variable rubber plantation conditions. This stability is consistent with the roles of MDE, ZOS-Mamba, and ALOC in improving crown-boundary discrimination, vertical structural continuity, and adaptive instance separation.

## 6 Discussion

Accurate individual-tree segmentation in densely planted rubber plantations is challenging because adjacent crowns often overlap and crown boundaries are visually and geometrically ambiguous. When only 3D geometric information is used, point-wise spatial features may be insufficient to distinguish neighboring crowns, especially in closed-canopy areas where crown edges are interlaced. To address this problem, the proposed MDA-SegNet introduces the MDE module to integrate 2D high-resolution visual cues with 3D LiDAR structural information. The 2D top-view information provides complementary boundary guidance, while the 3D point cloud preserves the vertical and spatial structure of individual trees. This multimodal design improves crown boundary discrimination and reduces the ambiguity caused by crown adjacency. The improved segmentation performance and the strong agreement in crown-related structural parameters indicate that combining visual boundary information with 3D geometry is effective for individual rubber tree crown segmentation in dense plantation environments.

While MDE mainly alleviates horizontal crown-boundary ambiguity, another important difficulty lies in maintaining vertical structural continuity within each tree instance. Rubber trees often show uneven point-density distributions between dense crown regions and relatively sparse trunk or lower-canopy regions. Conventional local geometric models may overemphasize dense crown features while failing to maintain feature continuity along the vertical direction, which can lead to incomplete trunk–crown association or fragmented tree instances. To address this issue, the ZOS-Mamba module combines Z-order spatial serialization with selective state-space modeling, enabling the network to capture both local spatial relationships and long-range structural dependencies along the vertical profile of the tree. This design helps preserve the structural integrity of individual trees from crown to stem, which is important for reliable tree height estimation and subsequent structural parameter extraction.

After improving horizontal boundary discrimination and vertical structural continuity, the next challenge is the morphological variability among rubber trees with different growth stages and management conditions. Rubber plantations may contain trees with different crown sizes, stem visibility, branching patterns, and canopy closure levels. Fixed-scale clustering strategies are often less effective under such variation because a single clustering scale cannot simultaneously fit young, mature, and densely planted trees. The ALOC module improves the adaptability of instance separation by introducing structure-aware adaptive clustering. Instead of relying on fixed clustering assumptions, ALOC adjusts instance association according to the local structural characteristics of the point cloud. The stable results across different Danzhou City blocks suggest that the proposed framework can better adapt to regional differences in rubber plantation structure and maintain consistent individual-tree segmentation performance.

The generalization experiments further show that the proposed framework is not limited to a single acquisition condition or forest structure. The FOR-instance dataset provides UAV-based LiDAR data from multiple forest regions, while NIBIO_MLS provides dense mobile laser scanning point clouds with different acquisition geometry and point-density characteristics. The performance on FOR-instance indicates that the model can be applied to UAV LiDAR forest scenes with different forest types and canopy structures. The additional results on NIBIO_MLS further show that the learned 3D structural representation remains effective under a different scanning platform. These results demonstrate that the proposed framework has good external adaptability across different point-cloud acquisition conditions. At the same time, the performance differences among subsets indicate that highly irregular forest structures, severe crown interlacing, and heterogeneous branching patterns remain difficult cases for individual-tree segmentation.

This external adaptability is important for practical UAV-based rubber plantation monitoring, where large-area scenes usually contain massive point clouds and different stand structures. Direct whole-scene inference may be constrained by GPU memory, especially when high-density UAV LiDAR data are used. In practical applications, the framework can be implemented using a block-wise inference strategy, where large point-cloud scenes are divided into spatial blocks and processed sequentially. The instance predictions from adjacent blocks can then be merged according to spatial consistency and instance association. This strategy allows the model to process UAV LiDAR scenes with different spatial extents while keeping memory consumption controllable. Therefore, although the present experiments mainly evaluate segmentation performance on plot-level and public benchmark datasets, the model architecture and inference strategy are compatible with larger UAV LiDAR scenes commonly encountered in rubber plantation monitoring.

The practical value of this segmentation capability is reflected in its potential contribution to rubber forest resource investigation and yield-related structural analysis. In rubber plantations, parameters such as tree height, crown diameter, crown area, and crown volume are closely related to stand growth status, canopy development, light interception, biomass accumulation, and potential latex production capacity. Traditional field surveys are labor-intensive and difficult to apply frequently over large plantation areas. By combining UAV LiDAR data and multimodal deep learning, MDA-SegNet provides a non-destructive and efficient alternative for extracting individual-tree structural information. Accurate individual-tree crown segmentation can support tree-level inventory, growth monitoring, stand structure assessment, and spatially refined plantation management. In particular, reliable crown and height parameters can provide useful inputs for yield prediction models, tapping management, and plantation productivity evaluation.

Nevertheless, translating these advantages into routine large-area rubber plantation monitoring still requires attention to several practical constraints. First, the current multimodal fusion strategy relies on the accurate co-registration of 2D imagery and 3D point clouds. Misalignment caused by wind-induced canopy movement, terrain variation, or sensor synchronization errors may introduce boundary noise. Second, although the model shows stable performance in rubber plantations and public forest point-cloud datasets, complex mixed forests with dense understory vegetation and highly irregular crown morphology still present challenges. Third, the current experiments mainly focus on structural segmentation and parameter extraction, while direct latex yield modeling requires multi-temporal observations and field production records. These issues indicate that further improvements in multimodal alignment, large-area deployment, and multi-temporal yield-related modeling are still needed to support more operational applications in rubber plantation management.

## 7 Conclusion

In this study, we present MDA-SegNet, a multimodal deep learning framework for individual-tree segmentation and structural parameter extraction in densely planted rubber plantations. The framework integrates 2D UAV imagery and 3D LiDAR point clouds to alleviate the limitations of single-modal geometric representations. The MDE, ZOS-Mamba, and ALOC modules are designed to address crown-boundary ambiguity, uneven vertical point-density distribution, and growth-stage-related structural variation, respectively.

Extensive experiments on our self-constructed dataset and public forest datasets demonstrate that MDA-SegNet outperforms state-of-the-art baselines. On the self-constructed RT-Set, MDA-SegNet achieved an mIoU of 76.58% and an F-score of 87.32%. Compared with the best-performing baseline RtSegNet, which achieved 76.16% mIoU and 86.80% F-score, MDA-SegNet improved these two metrics by 0.42 and 0.52 percentage points, respectively. This direct comparison more clearly demonstrates the advantage of the proposed framework over existing models in dense rubber plantation scenes. Furthermore, in the structural parameter extraction task, the model yielded accurate estimations for tree height, crown diameter, and crown volume, with *R*^2^ values reaching 0.97, 0.88, and 0.91, respectively. These results suggest that MDA-SegNet can provide reliable tree-level structural information for rubber plantation inventory, growth monitoring, and precision plantation management.

## Authorship contribution statement

**Weiqi Yin**: Conceptualization, Methodology, Validation, Visualization, Writing–original draft. **Jie Zhang**: Data curation, Investigation, Validation. **Hengrui Wang**: Data curation, Investigation. **Yuanyuan Zhang**: Writing–review & editing. **Jialei Zhan**: Writing–review & editing. **Jialang Liu**: Writing–review & editing. **Hui Lin**: Writing–review & editing. **Jiangquan Zeng**: Writing–review & editing. **Wentao Peng**: Writing–review & editing. **Guoxiong Zhou**: Resources, Funding acquisition. **Huaiqing Zhang**: Writing–review & editing, Funding acquisition, Project administration. **Xiangjun Wang**: Conceptualization, Writing–review & editing, Funding acquisition, Project administration.

## Funding

This research was supported by the Central Public-interest Scientific Institution Basal Research Fund (Grant No. 1630032022007), the 10.13039/501100004761Hainan Provincial Natural Science Foundation of China (Grant No. 324MS087), the National Key R&D Program of China (Grant No. 2019YFD1000500), and the Opening Project Fund of Key Laboratory of Biology and Genetic Resources of Rubber Tree, Ministry of Agriculture and Rural Affairs, P. R. China (Grant No. RRI-KLOF202305).

This work was also supported by the 10.13039/501100004735Hunan Provincial Natural Science Foundation Project (Grant No. 2025JJ50385) and the 10.13039/501100001809National Natural Science Foundation of China (Grant No. 62276276).

## Declaration of competing interest

The authors declare that they have no known competing financial interests or personal relationships that could have appeared to influence the work reported in this paper.

## Data Availability

Part of the datasets and the code used in this study are available in the GitHub repository at https://github.com/ywqgezi/MDA-SegNet. The remaining self-constructed datasets, including 36 sample plots in total, are available from the corresponding author upon reasonable request.
